# Sir Napier Burnett's Confidential Report

**Published:** 1920-10-02

**Authors:** 


### CUMBERLAND INFIRMARY INQUIRY.

### Sir Napier Burnett's Confidential Report.

Unusual interest was given to the last quarterly meeting of the governors of Cumberland Infirmary by the discussion which arose on the motion of Dr. Lediard that a copy of Sir Napier Burnett's recent report should be sent to each governor.

It should be explained that Sir Napier Burnett was lately asked by a sub-committee appointed after the annual meeting to hold an inquiry into the administration, in consequence of the receipt of a round robin from the nurses and probationers. The report was referred to a sub-committee, which refused to allow the report to be read by two representatives of nine governors who had previously sent in a requisition for a special meeting. This was never held, because the sub-committee had decided on the inquiry. Dr. Lediard's motion was opposed on the ground that the report was confidential, that its circulation might do harm to the infirmary, and in the end it was decided that the report should be available at the infirmary for interested governors only. Dr. Barnes presided over an attendance which included the Dean of Carlisle, Dr. Lediard, Dr. McLaren, Mr. Theodore Carrick, Mr. H. W. Sewell, Mr. E. W. Stead, Mr. Allan Hodgson, Mr. Joseph Irving, Mr. Reeves, Mr. J. P. D. Wheatley, Mr. G. Riddle, Mrs. James Carr, the Rev. E. Booth, and Mr. J. G. Howitt, secretary.

In support of his motion that Sir Napier Burnett's report be sent to each governor, Dr. Lediard explained that the small storm which was brewing at the annual meeting in February later came to a head. Twenty-seven nurses and probationers signed a round robin presenting their grievances. The Committee investigated these, and, after many meetings, made considerable concessions in respect of grievances which never should have existed in a well-managed hospital. These concessions, however, did not secure peace, and the Committee in search of an arbitrator secured Sir Napier Burnett, an expert on hospital management, who visited the infirmary, and held an inquiry which lasted for a day and a half. He interviewed nurses and other members of the staff, some governors from outside, and presented his report five weeks later. This report was full. It dealt not only with the nurses' grievances, but with the management, and contained various suggestions, some of which the financial state of the infirmary made impracticable. The report should be at the governors' disposal and could not be withheld. They were entitled by statute to see every letter, document, or report which was the property of the Committee or formed part of its business. The governors formed the supreme controlling authority, though they delegated some of their powers to the Committee. The management, money, and reputation of the infirmary were the governors' responsibilities, which they could not shirk. In support of his point that the governors had absolute power over all documents pertaining to the infirmary, Dr. Lediard referred to the regulations, from which he read. In his view, it would be a great mistake to withhold the report. He did not think its publication would harm the infirmary, whereas, if the report were withheld, the suspicion in the city in regard to the management of the infirmary would increase. The confidence was wanted of the general public, of all subscribers, and of every governor upon the subscription list.

Dr. Lediard's motion was seconded by Mr. Theodore Carrick and supported by Mr. H. W. Sewell, who said that the chief objection to the motion would be the cost involved in sending a copy of the report to everyone of the large number of governors. After careful thought he did not think that the issue of the report would injure the infirmary. It would be more likely to induce more governors to attend meetings and to take an active share in the management. It was felt that they objected to publicity, and he thought therefore that they should show that the feeling had no basis. If the report was not published, those who did not subscribe would have an excuse for holding aloof.

Mr. E. W. Stead said that he attached no importance to Sir Napier Burnett's report, which told them nothing that they did not know and made impracticable suggestions. He did not think it mattered in the least whether the governors were or were not familiar with the report. But publication would be expensive, and that was the chief objection to it. He would be perfectly willing to allow the governors to see the report, but he was opposed to the expense of its publication.

In answer to a question from the chairman, Dr. Barnes, Mr. Stead said that the cost of publication would be many pounds. Mr. Allan Hodgson opposed the motion because the report was made for the committee and intended to remain with the members. To send it to the three hundred governors would make it public property throughout the county. A public inquiry was held in regard to another hospital in England, and he did not think it did any good to that hospital. He did not question the right of the governors, but there was a great practical difference between a governor's inspection of a document in the infirmary and the placing of that document in his possession. After reading the report six times, he had come to the conclusion that it would be prejudicial to the infirmary to make it public. Many names were mentioned, and no one would be benefited by its publication.

The Dean of Carlisle declared that it would be "a fatal step" to publish the report broadcast. It was highly confidential, and he felt that Sir Napier Burnett would never have written it as he had done if he had supposed that it was destined to become public property. The report was concerned with matters strictly personal and reflected upon various persons, though it declared that the conduct of no official deserved his or her dismissal. The publication of the remarks concerning those mentioned would make their position in the hospital impossible. No institution could be conducted if every petty piece of friction, disagreement, or censure were published. The governors, like Parliament, were, as Dr. Lediard said, ultimately responsible, but the country could not go on if the proceedings of the Cabinet were published for the information of Parliament, and thus of the country. If the motion were adopted, it would amount almost to a vote of censure on the committee. He doubted if individual governors had the right to see the report. They had a right to see the official proceedings of the committee, but, in his view, no right to see such a document as this. He deprecated the suggestion that the report should be shown to any governor who asked for it. That would almost amount to its public circulation. Besides, publication might lead to legal proceedings.

Dr. Lediard : It is privileged.

The Dean : So long as it is shown to governors only.

Mr. Joseph Irving said that he supported Dr. Lediard because the public know about the trouble, and the

**Cumberland Infirmary Inquiry**—(*continued*).

infirmary was harmed thereby. Those whom he represented would subscribe handsomely to the appeal, but they had said that they would not do so until they knew the root of the trouble. Support was being withheld because the matter had never been cleared up.

Mr. G. Riddle said that if the committee would allow those governors who were interested to see the report, the governors had gained their point. If they could see the report they could judge for themselves the sufficiency of the committee's recommendations. They were in earnest, and reserved the right to call a special meeting of governors to have the matter thoroughly discussed.

Mr. Reeves said that the interested governors should see the report if only because he believed that they would then be satisfied with the action of the committee.

Mrs. Wheatley also argued that the governors who wished to see the report should be allowed to do so, and asked Dr. Lediard to withdraw his motion if this undertaking was given

Dr. Lediard said that he preferred to stand by his resolution. The expense of printing the report would not be great. The committee thought nothing of paying sixty guineas to Sir Napier Burnett, though he did not suggest that this was not deserved. When Mr. Allan Hodgson and Mrs. Carr interviewed the nurses, the whole business might have been settled. The effect had been to ruffle their feelings. Two stood out, the rest stood firm. It was a lost opportunity.

Mr. Riddle then moved that interested governors should be allowed to read the report at the infirmary. Mr. Theodore Carrick seconded the amendment, which was carried unanimously.

It was further decided that there should be attached to the report, before it was read by the governors, Sir Napier Burnett's accompanying letter, in which he said that he hoped that the report would be regarded as confidential.

The report of the Augmentation of Income Committee and the statement of accounts for the past quarter were then considered and adopted.

